# Study of the Relationship between Body Mass Index, Body Image, and Lifestyle Behaviors: A Community Survey in Fiji

**DOI:** 10.31662/jmaj.2019-0042

**Published:** 2019-11-08

**Authors:** Midori Ishikawa, Tetsuji Yokoyama, Nobuo Nishi, Hiroko Miura

**Affiliations:** 1 Department of Health Promotion, National Institute of Public Health, Wako, Japan; 2 International Center for Nutrition and Information, National Institute of Health and Nutrition, National Institutes of Biomedical Innovation, Health and Nutrition, Tokyo, Japan; 3 Department of International Health and Collaboration, National Institute of Public Health, Wako, Japan

**Keywords:** Overweight and obesity, body image, lifestyle behavior, underestimates, Fiji

## Abstract

**Introduction:**

Public health promotion efforts aimed at overweight and obesity prevention often proceed from the premise that the first step should be losing weight. Appropriate perceptions of body image may be important for improving weight loss awareness. Therefore, we aimed to examine the relationship between body mass index and body image perception in Fiji, where increasing obesity is currently the most important health-related issue.

**Methods:**

Using the resident register based on the governmental census, one household member, aged between 18 and 69 years, was randomly selected from each household in two areas. There were 1,014 participants in the study. The questionnaire items were related to body image, dietary behaviors, physical activity, smoking habits, and alcohol consumption habits. Moreover, information regarding height, weight, blood pressure, and hemoglobin A1c were measured, and questions about social status (age, ethnicity, education, marital status, and employment), and subjective living status were asked. A multivariate logistic regression analysis was performed to analyze the relationship between body mass index (BMI) and body image perception.

**Results:**

Data from 391 men and 537 women were analyzed. Men within higher BMI quartiles smoked less (p = 0.0004) and drank less alcohol (p = 0.042). Women in higher BMI quartiles engaged in less physical activity (p = 0.022). Among the assessed data, both men and women in the higher BMI quartiles underestimated their body image compared with their actual physique (p < 0.0001). The higher BMI was associated with underestimated body image (men: odds ratio [OR] = 3.22, 95% confidence interval [CI], 1.94–5.35; p < 0.0001; women: OR = 18.11, 95% CI, 10.10–32.47; p < 0.0001).

**Conclusions:**

Higher BMI is strongly associated with underestimated body image among Fiji residents. Health-related counseling should be included within programs that aim to increase recognition of one’s actual physical size.

## Introduction

Overweight and obesity represent a major public health problem and are associated with an increased risk for type 2 diabetes, cardiovascular disease, and several types of cancer ^(1), (2)^. Currently, obesity is a major public health issue in Fiji, as well as in other Pacific Island States, owing to the rapid lifestyle changes in the region, brought about by globalization and urbanization ^(3)^. An estimated 66.9% of the population is obese/overweight ^(4)^.

Public health promotion efforts aimed at weight gain and obesity prevention are often based on the assumption that raising awareness of health behaviors and weight status will motivate individuals to lose weight ^(4), (5), (6)^. However, an accurate perception of body image may be more important in successfully implementing these recommendations for controlling weight ^(6), (7)^.

Several reports indicate that obese people underestimate their own body image perception. Studies conducted in the US have observed that a high proportion of individuals who perceived themselves to be of normal weight were actually overweight or obese ^(8), (9), (10)^. In a US nationally representative study, 13% and 48.1% of obese and overweight men, respectively and 5.1% and 23.4% of obese and overweight women, respectively, believed they were “the right weight” ^(11)^.

Health guidance and education for obesity prevention and control emphasize weight monitoring with behavior changes related to diet and physical activity habits and provide instructions, such as on smoking and drinking habits. Moreover, adding guidance on body image perception may be important ^(12)^.

However, the guidelines on obesity prevention and control established by Fiji’s Ministry of Health and Medical Service (MOHMS) do not include the consistently required measures for those who underestimate their body image ^(13)^. Furthermore, a few reports state that an underestimation of body image compared with people’s true physique is important, including the relation between diet and physical activity. Therefore, we aimed to examine the relationship between obesity, body image perception, and lifestyle behaviors such as diet and physical activity.

## Materials and Methods

### Study population

All households in the target village were visited based on the resident register obtained from the governmental census, and the members of each family in Baulevu (rural area) and Nuffield (urban area), in the central division of Fiji, were checked. Next, a list of residents list aged between 18 and 69 years was created. Among the 1,927 households initially elected, 100 households were found to live away from the survey area, and another fifty-five households either did not have a member in the age range of 18–69 years or could provide a correct list of ages of the household members. Afterwards, one household member was randomly selected from each household to be surveyed. The number of men and women selected was the same by age group.

Invitation letters were distributed to the sampled household members. The survey was conducted in February, March, and May 2016, for those who agreed with the study’s objective. The survey took place at a designated area in the vicinity of their homes. Thus, one member from each of the 1,014 households among the 1,772 households participating in the study (participation rate: 57.2%) was invited. Data obtained from 391 men and 537 women were analyzed after excluding pregnant women and respondents who did not answer necessary items (Supplement).

### Measurements

The questionnaire items included indicators of existing policy measures and MOHMS guidelines in Fiji. Reports from the World Health Organization (WHO) and previous studies related to education for weight control were used ^(13), (14), (15), (16)^.

Body image perception (underestimate, appropriate. or overestimate) was described using SHOWCARD and the body mass index (BMI) ^(17), (18)^. Anthropometric measurements, comprising height and weight, were performed on all participants by health staff trained in the use of the stadiometer (Seca 213 Lightweight & Portable Stadiometer, Seca Deutschland, Germany), weight scale (Seca Clara 803 for normal weight, Seca 877 for larger weight, Seca Deutschland, Germany), and measuring tape. The anthropometric measurement procedures followed the WHO STEP guidelines ^(15)^.

Blood pressure was measured three times within three-minute intervals using a blood pressure monitor (Mediscope CONTEC08D, Mediscope International Limited, New Zealand). The average of the second and third measurements was used for the analysis. In addition, each participant was asked to provide venous blood, and 2 ml blood was taken from the forearm by a certified phlebotomist for testing HbA1c. HbA1C (g/dL) was measured using the latex particle. All samples were tested at Vanua Levu Medical Diagnostics Ltd. This sample was extracted by trained laboratory technicians. Laboratory technicians were responsible for the handling, storage, and delivery of the blood samples, with strict adherence to proper procedures to avoid contamination, as stipulated in the laboratory protocol.

The diagnostic criteria for diabetes and hypertension used the MOHMS in Fiji guidelines ^(13)^. Diabetes mellitus (DM) was defined as HbA1c ≥ 6.5% or controlled by medication, and hypertension (elevated blood pressure) was defined as blood pressure ≥140/90 mmHg or controlled by medication. In this study, the HbA1c level was divided into three categories: <5.6%, 5.6–6.5%, and >6.5%. In addition, the blood pressure level was divided into three categories: “Hypertension” was ≥140/90 mmHg or controlled by medication, “pre-hypertension” was systolic blood pressure (SBP)120–140 mmHg or diastolic blood pressure (DBP) 80–90 mmHg), and “normal” was SBP < 120 mmHg and DBP < 80 mmHg.

Social and health demographics accounted for within the sample were as follows: age; ethnicity (iTaukei, Fijian of Indian descent, other descent); education (primary school completed or less, secondary school completed, college/university completed or higher); marital status (never married, currently married, other); main work in the past 12 months (housewife, office worker, professional, service industry, student, farmer/fisherman/forester); and subjective living status (wealthy, very comfortable, reasonably comfortable, just getting along or less).

In lifestyle behaviors, at the stage of dietary behaviors, respondents were asked, “Are you going to change your dietary habits to prevent non-communicable diseases (NCDs)?” The answer was chosen from four categories: “I am not interested in change”; “I am going to change my dietary behavior in 6 months”; “I am ready to take action within the next 30 days”; “I have changed my dietary behavior relative to 6 months ago ^(19), (20)^.” At the stage of physical activity, respondents were asked, “Are you going to increase your physical activities to prevent NCDs?” The answer was chosen from four categories: “I am not interested in change”; “I am going to change my behavior in 6 months”; “I am ready to take action within the next 30 days”; “I changed my behavior more than 6 months ago ^(19), (20)^.” Moreover, smoking (currently: yes or no) and alcohol consumption in the past 12 months (daily; 5–6 days a week; 1–4 days a week; 1–3 days per month; less than once a month; never) were assessed ^(20)^.

### Statistical analyses

BMI was calculated as weight (kg)/height (m)^2^ and categorized into quartiles (25th, 50th, 75th, and maximum percentiles). Next, characteristics and social and health status were compared by BMI quartile using the chi-square test. In addition, lifestyle behaviors (dietary habits, physical activity, smoking habits, and alcohol consumption habits) were compared by BMI quartile using the chi-square test. Furthermore, the proportion of body image perception (underestimate, appropriate, and overestimate) by BMI quartile was analyzed using the chi-square test in males and females.

After that, to analyze the clear relationship between underestimation and BMI, participants who tended to overestimate their body mass were excluded from the next analysis. Then, a multivariate logistic regression analysis was performed. In BMI quartile, the first, second, third, and fourth BMI (i.e., the dependent variable) quartiles were assigned values of Q1 and Q2 = 1 and Q3 and Q4 = 0. The higher BMI category (Q3 and Q4) was the dependent variable. Model 1 was adjusted for age, ethnicity, education, marital status, and work; and Model 2 was adjusted for age, ethnicity, education, marital status, work, subjective living status, categories of HbA1c levels, and categories of blood pressure levels.

Statistical analysis was performed using SAS software, version 9.4 (SAS Institute, Inc., Cary, NC, USA). A p-value < 0.05 was considered statistically significant.

### Ethical aspects

The survey was conducted by Fiji’s MOHMS, and written consent was obtained from all participants. The analysis was submitted to the National Institutes of Public Health and National Institute of Health and Nutrition, National Institutes of Biomedical Innovation, Health and Nutrition, Japan, by Fiji’s MOHMS.

All personal information was omitted from the survey data, and the database obtained from Fiji’s MOHMS contained no personal information. This study was approved by the ethics board of the National Institute of Public Health, Japan, in June 2016 (NIPH-IBRA#12117).

## Results

Table 1 shows the demographic characteristics and social and health factors: age, ethnicity, education, marital status, main work, subjective living status, and diagnosis of DM and hypertension by BMI quartile. For males, the higher BMI quartile was related to many iTaukei (p < 0.0001), married individuals (p = 0.008), and those with hypertension (p < 0.0001). For females, those in the higher BMI quartile were older (p = 0.002), iTaukei (p < 0.0001), married (p = 0.035), and had hypertension (p < 0.0001).

**Table 1.** Demographic Characteristics and Social and Health Status by BMI Quartile.

			Male (n = 391)									Female (n = 537)								
			Q1 14.5 ≤ bmi < 22.8		Q2 22.8 ≤ bmi < 26.2		Q3 26.2 ≤ bmi < 30.3		Q4 30.3 ≤ bmi ≤ 52.3			Q1 13.9 ≤ bmi < 25.6		Q2 25.6 ≤ bmi < 30.1		Q3 30.1 ≤ bmi < 33.8		Q4 33.8 ≤ bmi ≤ 61.1		
			n 97	%	n 97	%	n 100	%	n 97	%	*p*-value	n 131	%	n 137	%	n 131	%	n 138	%	*p*-value
Age class																				
	18–29		30	30.9	27	27.8	20	20.0	12	12.4	0.350	50	38.2	28	20.4	22	16.8	20	14.5	0.002
	30–39		21	21.7	22	22.7	24	24.0	23	23.7		27	20.6	37	27.0	33	25.2	35	25.4	
	40–49		19	19.6	19	19.6	25	25.0	26	26.8		18	13.7	24	17.5	23	17.6	29	21.0	
	50–59		16	16.5	16	16.5	19	19.0	24	24.7		26	19.9	28	20.4	40	30.5	37	26.8	
	60–69		11	11.3	13	13.4	12	12.0	12	12.4		10	7.6	20	14.6	13	9.9	17	12.3	
Ethnicity																				
	iTaukei		34	35.1	53	54.6	56	56.0	73	75.3	<.0001	62	47.3	89	65.0	84	64.1	111	80.4	<.0001
	Fijian of Indian descent		62	63.9	40	41.2	40	40.0	20	20.6		68	57.9	47	34.3	42	32.1	22	15.9	
	Fijian of other descent		1	1.0	4	4.1	4	4.0	4	4.1		1	0.8	1	0.7	5	3.8	5	3.6	
Education																				
	Primary school completed or less		53	54.6	43	44.33	35	35.0	39	40.2	0.091	58	44.3	57	41.6	64	48.9	59	42.8	0.893
	Secondary school completed		33	34.0	36	37.1	40	40.0	35	36.1		53	40.5	59	43.1	52	39.7	57	41.3	
	College/University completed or higher		11	11.3	18	18.6	25	25.0	23	23.7		20	15.3	21	15.3	15	11.5	22	15.9	
Marital status																				
	Never married		37	38.1	21	21.7	21	21.0	15	15.5	0.008	27	20.6	11	8.0	12	9.2	15	10.9	0.035
	Currently married		57	58.8	70	72.2	70	70.0	73	75.3		89	67.9	106	77.4	96	73.3	100	72.5	
	Others		3	3.1	6	6.2	6	9.0	9	9.3		15	11.5	20	14.1	23	17.6	23	16.7	
Main work in the past 12 months																				
	Farmer		31	32.0	27	27.8	15	15.0	16	16.5	0.114	1	0.8	0	0.0	2	1.5	0	0.0	<.0001
	Fisher		0	0.0	2	2.1	1	1.0	2	2.1		0	0.0	0	0.0	1	0.8	0	0.0	
	Forestry		2	2.1	3	3.1	0	0.0	1	1.0		0	0.0	0	0.0	0	0.0	0	0.0	
	Office worker		4	4.1	0	0.0	7	7.0	5	5.2		5	3.8	3	2.2	2	1.5	9	6.5	
	Professional		8	8.3	10	10.3	12	12.0	14	14.4		2	1.5	6	4.4	4	3.1	9	6.5	
	Service industry		13	13.4	12	12.4	22	22.0	17	17.5		4	3.1	5	3.7	3	2.3	6	4.4	
	Student		4	4.1	7	7.2	3	3.0	1	1.0		20	15.3	2	1.5	1	0.8	4	2.9	
	Housewife		0	0.0	0	0.0	0	0.0	1	1.0		81	61.8	86	62.8	92	70.2	77	55.8	
	Other		26	26.8	26	26.8	33	33.0	30	30.9		9	6.9	28	20.4	19	14.5	26	18.8	
	No occupation		9	9.3	10	10.3	7	7.0	10	10.3		9	6.9	7	5.1	7	5.3	7	5.1	
Subjective living status																				
	Wealthy or Very comfortable		16	16.5	18	18.6	25	25.0	21	21.7	0.806	35	26.7	31	22.6	25	19.1	28	20.3	0.624
	Reasonably comfortable		47	48.5	49	50.5	45	45.0	48	49.5		46	35.1	60	43.8	58	44.3	56	40.6	
	Just getting along or less		34	35.1	30	30.9	30	30.0	28	28.9		50	38.2	46	33.6	48	36.6	54	39.0	
Nutritional status																				
	Categories of HbA1c level, % †																			
		HbA1c < 5.6	61	62.9	62	63.9	60	60.0	64	66.0	0.377	88	67.2	98	71.5	83	63.4	86	62.3	0.143
		5.6 ≤ HBb1c < 6.5	26	26.8	18	18.6	19	19.0	20	20.6		24	18.3	24	17.5	22	16.8	36	26.1	
		6.5 ≤ HbA1c or medication	10	10.3	17	17.5	21	21.0	13	13.4		19	14.5	15	11.0	26	19.9	16	11.6	
	Categories of blood pressure level, mmHg ‡																			
		Normal	53	54.6	32	33.0	19	19.0	11	11.3	<.0001	88	67.2	49	35.8	33	25.2	23	16.7	<.0001
		Pre-hypertension	38	39.2	55	56.7	62	62.0	60	61.9		34	26.0	69	50.4	70	53.4	65	47.1	
		Hypertension	6	6.2	10	10.3	19	19.0	26	26.8		9	6.9	19	13.9	28	21.4	50	36.2	

Data are expressed as number and percent (%). The *p*-values were calculated using chi-square test. † Categories of HbA1c levels: HbA1c ≥ 6.5 or controlled by medication; 6.5 > HBb1c ≥ 5.6; 5.6 > HBb1c ‡ Categories of blood pressure levels: Hypertension; SBP ≥ 140 and DBP ≥ 90 or controlled by medication, Pre-hypertension; 140 > SBP ≥ 120 or 90 > DBP ≥ 80, Normal; 120 > SBP and 80 > DBP

Table 2 shows lifestyle behaviors: diet, physical activity, smoking, and alcohol consumption by BMI quartile. For males, the higher BMI quartile smoked less (p = 0.0004) and had lower alcohol consumption rates (p = 0.042). For females, those within higher BMI quartiles indicated a lower frequency of physical activity (p = 0.022).

**Table 2.** Lifestyle Behaviors by BMI Quartile.

		Male (n = 391)									Female (n = 537)								
		Q1 14.5 ≤ bmi < 22.8		Q2 22.8 ≤ bmi < 26.2		Q3 26.2 ≤ bmi < 30.3		Q4 30.3 ≤ bmi ≤ 52.3			Q1 13.9 ≤ bmi < 25.6		Q2 25.6 ≤ bmi < 30.1		Q3 30.1 ≤ bmi < 33.8		Q4 33.8 ≤ bmi < 61.1		
		n 131	% 24.4	n 137	% 25.5	n 133	% 24.4	n 138	% 25.7	*p*-value	n 131	% 24.4	n 137	% 25.5	n 131	% 24.4	n 138	% 25.7	*p*-value
Dietary habit (stage)																			
	I am not interested in change	6	6.2	0	0.0	4	4.0	5	5.2	0.458	6	4.6	6	4.4	3	2.3	6	4.4	0.087
	I am going to change the behavior in 6 months	8	8.3	11	11.3	14	14.0	12	12.4		17	13.0	26	19.0	15	11.5	13	9.4	
	I am ready to take action within the next 30 days	58	59.8	62	63.9	62	62.0	54	55.7		74	56.5	84	61.3	85	64.9	99	71.7	
	I have changed the behavior more than 6 months ago	25	25.8	24	24.7	20	20.0	26	26.8		34	26.0	21	15.3	28	21.4	20	14.5	
Physical activity habit (stage)																			
	I am not interested in change	7	7.2	0	0.0	3	3.0	5	5.2	0.179	5	3.8	3	2.2	3	2.3	7	5.1	0.022
	I am going to change the behavior in 6 months	7	7.2	10	10.3	17	17.0	11	11.3		18	13.7	23	16.8	16	12.2	17	12.3	
	I am ready to take action within the next 30 days	57	58.8	61	62.9	59	59.0	58	59.8		75	57.3	89	65.0	85	64.9	103	74.6	
	I have changed the behavior more than 6 months ago	26	26.8	26	26.8	21	21.0	23	23.7		33	25.2	22	16.1	27	20.6	11	8.0	
Smoking (currently)																			
	Yes	55	56.7	42	43.3	39	39.0	26	26.8	0.0004	15	11.5	18	13.1	16	12.2	22	15.9	0.714
	No	42	43.3	55	56.7	61	61.0	71	73.2		116	88.6	119	86.9	115	87.8	116	84.1	
Alcohol consumption (past 12 months)																			
	Dairy	7	7.2	1	1.0	1	1.0	2	2.1	0.042	1	0.8	0	0.0	0	0.0	0	0.0	0.724
	5–6 days a week	1	1.0	0	0.0	3	3.0	1	1.0		0	0.0	0	0.0	0	0.0	0	0.0	
	1–4 days a week	9	9.3	9	9.3	14	14.0	7	7.2		2	1.5	3	2.2	2	1.5	3	2.2	
	1–3 days per month	21	21.7	23	23.7	16	16.0	12	12.4		7	5.3	6	4.4	5	3.8	11	8.0	
	Less than once a month/never	59	60.8	64	66.0	66	66.0	75	77.3		121	92.4	128	93.4	124	94.7	124	81.0	

Data are expressed as number and percent (%). The *p*-values were calculated using chi-square test.

**Figure 1.**
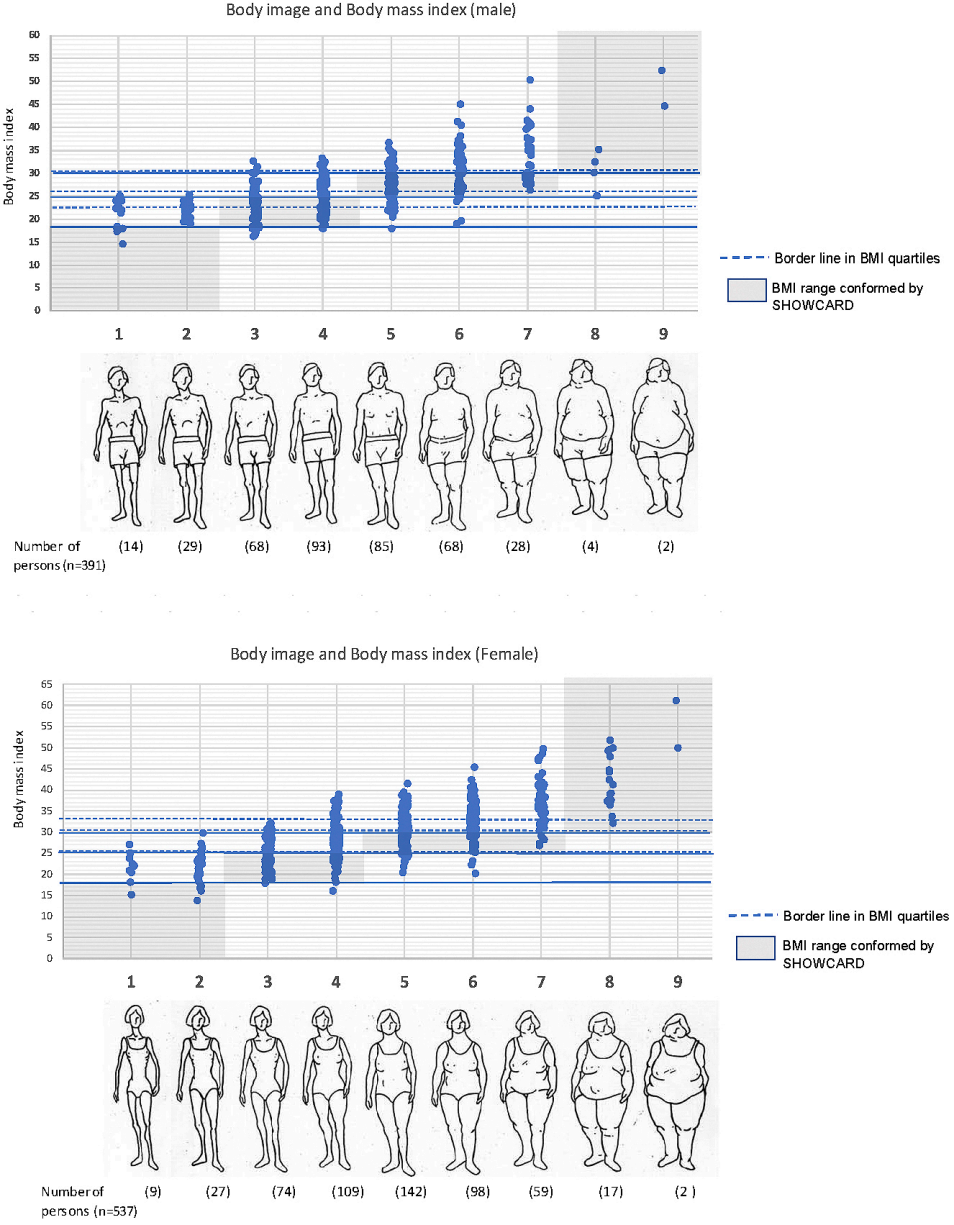
Relationship between body mass index and body image perceptions in males and females Body image and Body mass index. ……Border line in BMI quartiles □BMI range conformed by SHOWCARD.

Figure 1 shows the relationship between BMI and body image perceptions for both male and female subjects. Those who were overweight and obese (BMI) underestimated their actual body size. This result was true for both male and female subjects.

**Figure 2.**
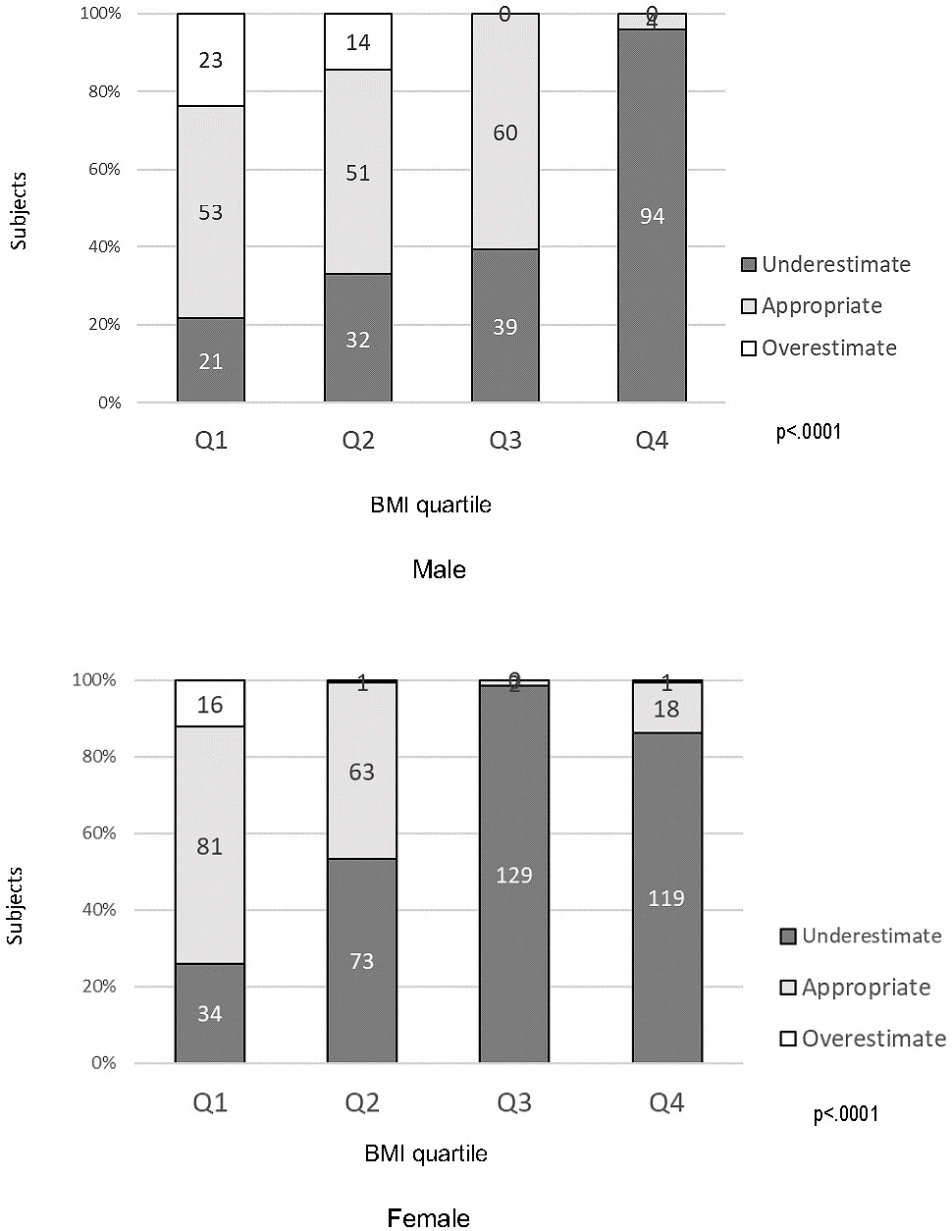
Proportion of body image perception by BMI quartile in males and females BMI quartile and Subjects. Data are expressed as the proportion of body image perception (underestimate, appropriate and overestimate) by BMI quartile. The *p*-values were calculated using chi-square test.

Figure 2 shows the proportion of body image perception by BMI quartile in both males and females. Whether the body image perception (underestimate, appropriate, or overestimate) was comparable with the actual physique (BMI < 18.5 = underweight, 18.5 ≤ BMI < 25.0 = normal, 25.0 ≤ BMI < 30.0 = overweight, BMI ≤ 30.0 = obesity) was confirmed. In terms of excess weight and obesity, both men and women underestimated their body image when compared with their actual BMI (p < 0.0001). Moreover, sub-analyzes indicated this phenomenon occurred more often among older obese men than young men (p = 0.011).

Table 3 shows the factors in higher obesity related to body image and lifestyles behaviors in male. In Model 1, these factors included an underestimation of body image (odds ratio [OR] = 3.57; 95% confidence interval [CI]: 2.20–5.81; p < 0.001) and smoking habits (OR = 1.71; 95% CI: 1.04–2.82; p = 0.036). In Model 2, these factors included an underestimation of body image (OR = 3.22; 95% CI: 1.94–5.35; p < 0.001). Then, stepwise analysis was performed, and the result was same as Model 2.

**Table 3.** Factors in Higher Obesity Related to Body Image and Lifestyles Behaviors (Male).

			Model 1				Model 2			
			OR	95%CI		p-value	OR	95%CI		p-value
Perception of body image	Appropriate (no: underestimate)	yes	1.00				1.00			
		no	3.57	2.20	5.81	<.0001	3.22	1.94	5.35	<.0001
Dietary habit	Have changed behavior more than 6 months ago	yes	1.00				1.00			
		no	1.34	0.76	2.36	0.306	1.49	0.82	2.70	0.192
Physical activity	Have changed behavior more than 6 months ago	yes	1.00				1.00			
		no	1.46	0.83	2.56	0.190	1.61	0.89	2.93	0.116
Smoking	Currently smoke	yes	1.00				1.00			
		no	1.71	1.04	2.83	0.036	1.53	0.90	2.61	0.120
Alcohol consumption	Less than once a month/never past 12 months	yes	1.00				1.00			
		no	0.89	0.53	1.50	0.655	0.81	0.47	1.39	0.439

The higher BMI categories (Q3 and Q4) were the dependent variables. The factors that might affect BMI included perceptions of body image, dietary habit, physical activity, smoking habits, and alcohol consumption habits. Model 1: adjusted for age, ethnicity, education, marital status, work Model 2: adjusted for age, ethnicity, education, marital status, work, subjective living status, categories of HbA1c level, categories of blood pressure levels OR: odds ratio, CI: confidence interval

Table 4 shows the factors in higher obesity related to body image and lifestyle behaviors for females. In Model 1, these factors included an underestimation of body image (OR = 17.05; 95% CI: 9.78–29.71; p < 0.001). Moreover, in Model 2, these factors included an underestimation of body image (OR = 18.11; 95% CI: 10.10–32.47; p < 0.001). Then, stepwise analysis was performed, and the result was same as Model 2.

**Table 4.** Factors in Higher Obesity Related to Body Image and Lifestyle Behaviors (Female).

			Model 1				Model 2			
			OR	95%CI		p-value	OR	95%CI		p-value
Perception of body image	Appropriate (no: underestimate)	yes	1.00				1.00			
		no	17.05	9.78	29.71	<.0001	18.11	10.10	32.47	<.0001
Dietary habit	Have changed behavior more than 6 months ago	yes	1.00				1.00			
		no	1.21	0.75	1.95	0.435	1.22	0.74	2.02	0.431
Physical activity	Have changed behavior more than 6 months ago	yes	1.00				1.00			
		no	1.62	0.98	2.68	0.060	1.49	0.88	2.52	0.142
Smoking	Currently smoke	yes	1.00				1.00			
		no	1.15	0.66	2.00	0.628	1.05	0.58	1.90	0.864
Alcohol consumption	Less than once a month/none past 12 months	yes	1.00				1.00			
		no	1.46	0.70	3.06	0.317	1.60	0.74	3.50	0.235

The higher BMI categories (Q3 and Q4) were the dependent variables. The factors that might affect BMI included perceptions of body image, dietary habit, physical activity, smoking habits, and alcohol consumption habits. Model 1: adjusted for age, ethnicity, education, marital status, work Model 2: adjusted for age, ethnicity, education, marital status, work, subjective living status, categories of HbA1c levels, categories of blood pressure levels OR: odds ratio, CI: confidence interval

## Discussion

The present study suggests that obesity is strongly associated with underestimated body image in Fiji. Recent research found that cultural norms, ethnicity, and socioeconomic status influence the relationship between BMI and body image perception ^(21), (22), (23)^. Moreover, changes in status related to marriage, childbirth, and parenting can change the perception of body image ^(24)^. In another study, those who were overweight not only underestimated their body image but also had low concerns about the threat of obesity ^(25), (26)^.

According to this study’s results, besides ethnicity, body image underestimation was a factor related to obesity, regardless of whether the participant was married. Furthermore, another study reported differences in body image satisfaction and eating attitudes between rural and urban young adult females ^(12), (27)^. Although this study included both urban and rural areas, body image perception and ethnicity were related with obesity regardless of the area. Education and guidance, starting from a young age and based on ethnic characteristics and body image perceptions, are needed ^(28), (29)^. In the next study, we aim to identify the appropriate body image for each BMI quartile, with regard to ethnicity, and analyze the participants’ characteristics related to the perception of body image.

In the MOHMS of Fiji, the guidelines on obesity prevention and control do not include priority measures for those who underestimate their body image ^(13)^. However, since the importance of counseling in this population has been agreed upon ^(14)^, content geared toward an accurate assessment of one’s body image should be included ^(30), (31)^.

Our study has certain limitations. First, our sample size may be relatively small compared with other similar epidemiological studies conducted in developed countries, because we started with identifying all members of a family by visiting households. Therefore, although the target number was small, it was possible to select the target person from the accurate resident list.

Second, there were more women than men in the survey collaborators. Of the participants in this study, about sixty percent of women were housewives, while forty percent of men were farmers or workers in the service industry. Women may consider it easier to participate in a survey than men. In the future, it is necessary to reconsider the survey method for men.

Third, the households that did not participate in this survey were those with no one at home at the time, and their work place (e.g., farm or company) was far from their house. Despite these limitations, higher BMI was associated with underestimation of body image.

### Conclusions

Higher BMI is strongly associated with underestimated body image among Fiji residents. Health-related counseling should be included within programs that aim to increase the recognition of one’s actual physical size.

## Article Information

### Conflicts of Interest

None

### Sources of Funding

This work was supported by Grants-in-Aid for Scientific Research from the Japan Ministry of Health, Labour and Welfare (grant number H27 Global Scale/issue General 002).

The funding institution had no role in the study design, data collection, analysis and interpretation of results, or in the writing of the manuscript, or the decision to submit the paper for publication.

### Acknowledgement

This study was conducted as part of the Project for Prevention and Control of Non-Communicable Diseases under the Japan International Cooperation Agency (JICA) and Fiji’s Ministry of Health and Medical Service (MOHMS). The authors thank all of the staff of JICA and Fiji’s MOHMS.

### Author Contributions

Midori Ishikawa conceptualized and analyzed the study, drafted the initial manuscript, and approved the final manuscript as submitted.

Tetsuji Yokoyama supervised the statistical analyses, reviewed and revised the manuscript, and approved the final manuscript as submitted.

Nobuo Nishi conceptualized and designed the study, coordinated the data collection, reviewed and revised the manuscript, and approved the final manuscript as submitted.

Hiroko Miura supervised this study, critically reviewed and revised the manuscript, and approved the final manuscript as submitted.

### Approval by Institutional Review Board (IRB)

This study was approved by the ethics board of the National Institute of Public Health in Japan (NIPH-IBRA#12117).

## Supplement

Supplementary Material
