# Supplementary figures and images for: Study of the Relationship between Body Mass Index, Body Image, and Lifestyle Behaviors: A Community Survey in Fiji
Source: JMA J. 2019 Nov 8;3(1):41–50. doi: 10.31662/jmaj.2019-0042 (PMC7733744; doi:10.31662/jmaj.2019-0042)

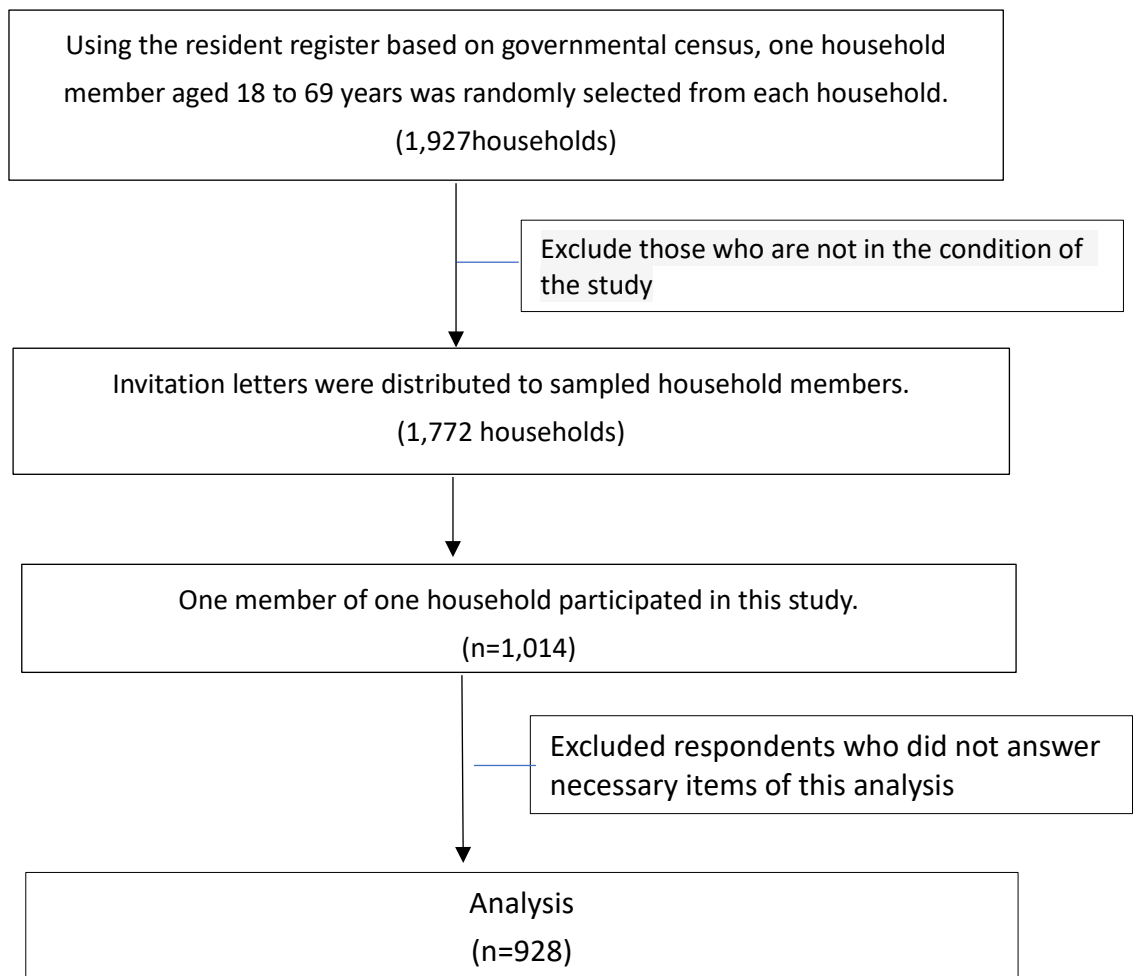

## Study population

Supplement: Supplementary file 1 — Supplementary Material [file 2433-3298-3-1-0041-s001.pdf]
